# Preoperative pulmonary ultrasound: a valuable tool for managing post-COVID-19 sequelae

**DOI:** 10.1016/j.bjane.2025.844697

**Published:** 2025-10-29

**Authors:** Luis Alberto Rodrigues Linares, Victória Regina da Silva Oliveira, Lais Helena Navarro e Lima, Rodrigo Moreira e Lima, Camila Squarzoni Dale

**Affiliations:** a Hospital São Luiz Rede Dor, Departamento de Anestesiologia, São Paulo, SP, Brazil; b Instituto de Ciências Biomédicas da Universidade de São Paulo, Departamento de Anatomia, São Paulo, SP, Brazil; c Perioperative and Pain Medicine of Manitoba University, Department of Anesthesiology, Winnipeg, MB, Canada; d Faculdade de Medicina de Botucatu da Universidade Estadual Paulista “Júlio de Mesquita Filho” (UNESP), Departamento de Anestesiologia e Especialidades Cirúrgicas, Botucatu, SP, Brazil

**Keywords:** SARS-CoV-2, Lung ultrasound, Perioperative Care

Dear Editor,

Lung Ultrasound (LUS) has emerged as a valuable diagnostic tool for evaluating residual lung injuries in patients recovering from SARS-CoV-2 infection. While LUS has gained recognition in critical care and emergency settings over the past two decades, its full potential as a preoperative risk assessment tool, especially in post-COVID-19 patients, remains largely unexplored. Originally described by Lichtenstein et al. in 1997 for the detection of alveolar-interstitial syndrome via ultrasound artifacts like the comet-tail sign, LUS has evolved into a cornerstone of pulmonary imaging in the Intensive Care Unit (ICU).^1^ Its appeal lies in its non-invasiveness, bedside applicability, absence of radiation, and low cost, demonstrating its superiority over physical examination and conventional chest X-Ray in detecting pleural and parenchymal abnormalities.^2^^,^^3^

The emergence of portable ultrasound devices has further enabled its application in various settings ‒ from operating rooms to pre-hospital environments. Despite these advantages, the integration of LUS into the routine practice of non-radiologist physicians is limited, often due to a lack of training and institutional barriers to access.^4^ With the advent of the COVID-19 pandemic, the need for point-of-care imaging became more pressing than ever. In just the initial months of the pandemic, millions were infected, and tens of thousands of lives were lost globally. Brazil was among the severely affected nations, reporting over 40,000 confirmed cases and more than 2500 deaths within the first months of 2020,^5^ which have grown exponentially to the present days. The virus posed not only an acute challenge to global health systems but also left a growing population of patients with persistent pulmonary complications, whose long-term management is still being defined.

It is well established that chest CT imaging in COVID-19 patients reveals typical peripheral and bilateral lung lesions, often described as “ground-glass” opacities. These findings are most frequently located in the posterior and lower lobes. While CT remains the gold standard for diagnosing such lesions, LUS has proven to be a reliable, real-time, bedside alternative, particularly for detecting superficial, pleura-associated abnormalities seen in Acute Respiratory Distress Syndrome (ARDS) and COVID-19.^6^^,^^7^

Our prospective observational study aimed to detect persistent pulmonary sequelae in post-COVID-19 patients during the convalescence phase and to assess their potential impact on preoperative risk stratification. Therefore, 31 adult patients recovering from SARS-CoV-2 infection were evaluated. All patients included in the study agreed and signed a written informed consent form (CAAE: 37,658,720.4.0000.0087). The patients underwent lung ultrasound between 4 and 6 weeks after symptom onset as part of their post-recovery assessment. All had been hospitalized, and many had required intensive care. We specifically excluded mild COVID-19 cases and those with pre-existing chronic lung disease to isolate findings associated with acute SARS-CoV-2 infection. Lung ultrasound was conducted using a standardized image acquisition protocol recommended by the Italian LUS-COVID expert team.^8^ Their lungs were evaluated by a physician with at least three years of experience performing focused LUS. Fourteen lung regions (three posterior, two lateral and two anterior) were examined for 10 s each per patient using either convex or linear probes, and images were scored based on pleural line appearance and presence of B-lines or consolidations (Figure 1).

**Figure 1:**
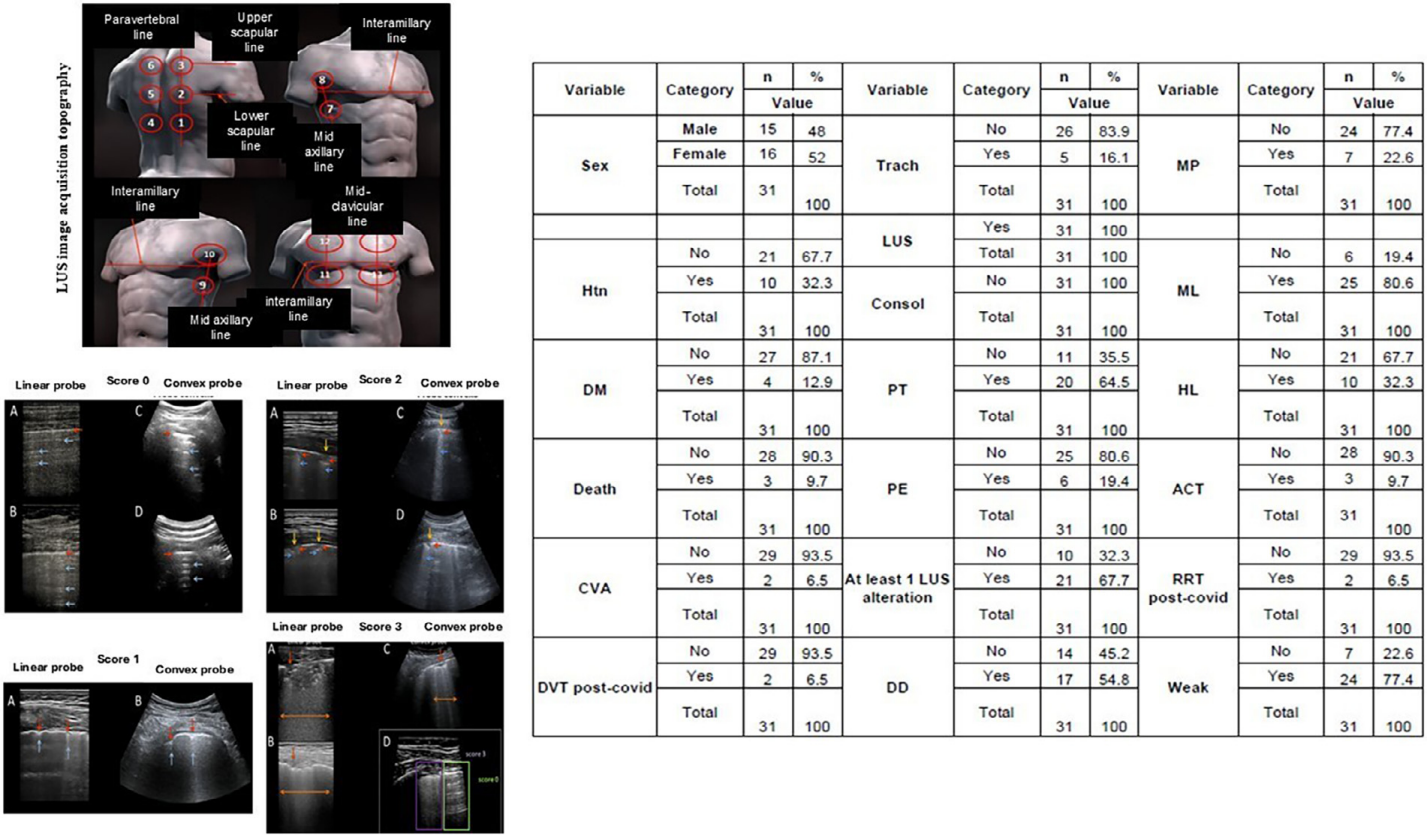
**Epidemiological and clinical history data.** Qualitative data of 31 participants were evaluated; data were presented as percentage ( %). LUS images for each score, obtained with a linear probe (A) and a convex probe (B). Score 0: continuous and regular pleural line (red arrows); horizontal artifacts – A lines (blue arrows). Score 1: indented pleural line (red arrows); sparse B lines present (blue arrows). Score 2: pleural line with interruptions (yellow arrows); below the pleural line interruption points, small areas of consolidation are present (red arrows), associated with areas of coalescent vertical artifacts (B lines) (blue arrows). Score 3: pleural line with extensive interruptions; Below the points of discontinuity of the pleura, extensive pulmonary consolidations can be found (red arrows), associated with generalized areas of “white lung” (orange arrows). Htn, Hypertension; DM, Diabetes Mellitus; CVA, Cerebral Vascular Accident; DVT, Deep Vein Thrombosis; Trach, Tracheostomy; LUS, Lung Ultrasound; Consol, Consolidation; PT, Pleural Thickening; PE, Pleural Effusion; DD, Depression disorder; MP, Muscle Pain; ML, Memory Loss; HL, Hair Loss; ACT, Anticoagulation; RRT, Renal Replacement Therapy; Weak, Body Weakness.

The results showed that 100 % of patients had some degree of lung consolidation, and 67.7 % exhibited abnormalities scored as 2 or 3. The most frequent findings included pleural thickening (64.5 %) and pleural effusion (19.4 %). These structural changes were detected well into the convalescence period and affected not only the clinical perception of recovery but also preoperative risk. In addition to pulmonary alterations, a significant portion of patients reported lingering emotional and physical sequelae, including depression (54.8 %), memory loss (80.6 %), muscle weakness (77.4 %), and hair loss (32.3 %) (Figure 1). Notably, three patients died following their post-COVID recovery period despite having undergone LUS evaluation beforehand. These outcomes underscore the critical need for robust perioperative risk stratification tools in this population.

Although CT imaging remains superior in terms of anatomical resolution, its practical limitations ‒ radiation exposure, cost, lack of portability ‒ make it less ideal for bedside risk assessment prior to surgery. LUS, on the other hand, provides dynamic, real-time insights into lung aeration, interstitial involvement, and pleural integrity. Importantly, it can be conducted by trained clinicians in non-radiology specialties, expanding its utility in both inpatient and outpatient settings.

Currently, no widely adopted protocols incorporate lung ultrasound into preoperative evaluations of post-COVID-19 patients. Our study supports the argument that they should be included. Approximately two-thirds of patients in our cohort exhibited persistent pulmonary abnormalities detectable via LUS, so anesthesiologists and surgical teams would benefit from incorporating this tool into standard evaluation protocols. Doing so could allow for individualized ventilation strategies, perioperative respiratory therapy, and fluid management, ultimately improving outcomes. We also note that interobserver agreement in LUS interpretation was high in our study, affirming that with appropriate training, the technique yields reproducible and clinically relevant results. Such reliability bolsters its potential for broader adoption across healthcare teams.

In conclusion, LUS is a promising, underutilized modality for detecting pulmonary sequelae in post-COVID-19 patients. It is simple, affordable, and can be performed at the bedside without the logistical and financial burden of more complex imaging.^9^ Given the persistent and often underestimated respiratory complications in COVID-19 survivors, particularly those requiring ICU admission, the incorporation of lung ultrasound into preoperative assessments may represent an important evolution in perioperative care. We urge healthcare institutions and surgical teams to consider routine use of lung ultrasound in post-COVID patients, particularly those undergoing procedures requiring anesthesia. Future multicenter studies should explore the correlation between LUS findings and postoperative complications to further validate its role in perioperative medicine.

## Data availability statement

The datasets generated and/or analyzed during the current study are available from the corresponding author upon reasonable request.

## Authors’ contributions

LARL, VRSO, CSD: Wrote and prepared the manuscript. LARL, LHNL, RML: Conceptualized and collected data.

## Conflicts of interest

The authors declare no conflicts of interest.
